# Withdrawal of antihypertensive medication in young to middle-aged adults: a prospective, single-group, intervention study

**DOI:** 10.1186/s40885-022-00225-2

**Published:** 2023-01-02

**Authors:** Hae-Young Lee, Kyoung Suk Lee

**Affiliations:** 1 Department of Internal Medicine, Seoul National University Hospital, Seoul, Republic of Korea; 2 Research Institute of Nursing Science, Center for Human-Caring Nurse Leaders for the Future by Brain Korea 21 (BK 21) Four Project, Seoul National University College of Nursing, Seoul, Republic of Korea

**Keywords:** Hypertension, Antihypertensive agents, Deprescriptions

## Abstract

**Background:**

Although antihypertensive drug therapy is commonly believed to be a life-long therapy, several recent guidelines have suggested that antihypertensive medications can be gradually reduced or discontinued for some patients whose blood pressure (BP) is well-controlled for an extended period. Thus, this pilot study aimed to describe the success rate of antihypertensive drug discontinuation over 6 months among young and middle-aged patients with hypertension.

**Methods:**

This was a prospective, single-group, intervention study. Patients were eligible for inclusion if their cardiologist judged them to be appropriate candidates for this study, their BP had been controlled both in the office (< 140/90 mmHg) and 24-h ambulatory BP monitoring (< 135/85 mmHg) for at least 6 months with a single tablet dose of antihypertensive medication. A total of 16 patients withdrew their antihypertensive medications at baseline after they received the education, and were followed up over 6 months. After the follow-ups, six patients participated in the in-depth interview.

**Results:**

The likelihood of remaining normotensive at 30, 90, 180, and 195 days was 1.00, 0.85, 0.51, and 0.28, respectively. There were also no significant differences in baseline characteristics and self-care activities over time between normotensive (*n* = 8) and hypertensive groups (*n* = 8). In the interview, most patients expressed ambivalent feelings toward stopping medications. Psychological distress (e.g., anxiety) was the primary reason for withdrawal from this study although the patients’ BP was under control.

**Conclusions:**

We found that only a limited portion of antihypertensive patients could stop their medication successively over 6 months. Although we could not identify factors associated with success in maintaining BP over 6 months, we believe that careful selection of eligible patients may increase success in stopping antihypertensive medications. Also, continuous emotional support might be essential in maintaining patients’ off-medication.

## Background

Hypertension is a prevalent chronic condition that significantly contributes to cardiovascular disease and premature death [1, 2]. Lifestyle modifications and antihypertensive medication are the major components of hypertension management [3–5]. According to the 2018 guidelines of the Korean Hypertension Society [5], drug treatment is initiated when the systolic/diastolic blood pressure (BP) is ≥ 140/90 mmHg, along with lifestyle modifications. Although antihypertensive medications are very effective in BP lowering, some patients refuse it or are nonadherent to the treatment. According to a recent meta-analysis, the overall nonadherence rate to antihypertensive medications was 45.2% [6]. The common reasons for refusal or nonadherence to taking antihypertensive drugs are related to concerns about the side effects of the medications, social stigma, and the burden of taking drugs for the rest of their lives once they start medications for hypertension [7–10]. One of the most frequently asked questions from the patients who need drug treatment is whether they should take antihypertensive medication indefinitely once it is started is.

Although antihypertensive drug therapy is commonly believed to be a life-long therapy, several recent guidelines [4, 5] have suggested that antihypertensive medications can be gradually reduced or withdrawn for some patients whose BP is well-controlled for an extended period. However, the level of evidence for this recommendation is very weak. A recent experimental study suggested that when antihypertensive medications were stopped from the patients at low-cardiovascular disease risk, BP control at the 2-year follow-up was not inferior to standard care [11]. However, the investigators did not explore the characteristics of patients who successfully stopped antihypertensive medications. In addition, in this study, it was optional for patients in the intervention group to consult with their clinicians regarding their medication withdrawal. About 35% of the patients in the intervention group did not attempt to have their medications withdrawn. However, the reasons were not investigated further in that study [11]. This result implies that some patients with low-cardiovascular disease risk continued taking their antihypertensive medications even though they were eligible to discontinue them. Another study showed far more pessimistic results. The short treatment with the angiotensin receptor blocker candesartan surveyed by telemedicine (STAR CAST) Study investigated whether treatment with candesartan or nifedipine-controlled release resulted in a sustained regression of hypertension in 244 patients with stage 1 essential hypertension [12]. After 1 year of treatment, the medications were tapered and discontinued. During the 1-year follow-up, there was a substantial reoccurrence of hypertension; at the study end, only one patient remained normotensive without antihypertensive medication [13]. Thus, it is important to explore who can successfully discontinue antihypertensive medications and the characteristics of patients who do not want to stop medications despite a low risk for cardiovascular disease. It is also essential to explore their reasons for such a decision.

The primary aims of this pilot study were to (1) describe the success rate of antihypertensive drug discontinuation over 6 months for young to middle-aged patients with hypertension; (2) to compare the changes in BP between the normotensive group (i.e., those who successfully discontinued their medications) and the hypertensive group over 6 months; and (3) compare the baseline sample characteristics and changes in adherence to self-care activities for hypertension between the normotensive and hypertensive groups. The secondary aims of this study were to describe the retention rate and the reasons for study withdrawal and compare the characteristics of patients who remained in the study and those who dropped out.

## Methods

### Study design and participants

This study was a prospective single-group interventional study and was approved by the Institutional Review Board of the Seoul National University Hospital (No. H-1902–132-1015). Patients were recruited from a cardiology clinic in Seoul National University Hospital, a tertiary academic medical center in Republic of Korea. Patients were eligible for inclusion if their cardiologist judged them to be appropriate candidates for this study, their BP had been controlled in normotensive range (< 140/90 mmHg) for at least 6 months with a single tablet dose of antihypertensive medication; the value of their 24-h ambulatory BP monitoring within 1 month before the study enrollment was 135/85 mmHg or below; they were between 20 to 60 years old. Patients with secondary hypertension, cardiovascular complications (e.g., myocardial infarction), or renal dysfunction were excluded.

### Outcome

The outcome of this study was the success rate of antihypertensive medication withdrawal, which was assessed from the cumulative time-related curve over 6 months. If patients met one or more of the following criteria, they were considered unsuccessful in discontinuing their antihypertensive medications, and were categorized in the hypertensive group: (1) office systolic BP of 140 mmHg above or diastolic BP of 90 mmHg above on two consecutive visits, (2) office systolic BP of 160 mmHg above or diastolic BP of 100 mmHg above on any one visit, or (3) resumed antihypertensive medication.

### Blood pressure measurement

The automated office BP readings were obtained based on standard procedures. The first BP readings were obtained in both arms, and then patients were instructed to use the arm with a higher BP monitoring throughout the study. Patients had a series of three BP readings at least 2 minutes apart after resting for at least 5 minutes. A mean of three consecutive automated office BP readings was recorded.

### Factors related to the success of antihypertensive medication discontinuation

#### Adherence to self-care activities for hypertension

Adherence to self-care activities for hypertension is based on the behavior subdomain of the Hypertension Self-Care Profile Behavior Scale [14]. The behavior subdomain consists of 20 items related to recommended lifestyle modifications (e.g., low fat and low sodium diet, exercise, smoking cessation, and stress management), taking medications as directed, and attending all clinic appointments. Each item is rated on a 1 to 4 Likert scale, and total scores can range from 20 to 80, with higher scores indicating better adherence to self-care activities. Because two items related to taking medications were irrelevant for this study, we used the remaining 18 items, with the total scores ranging from 18 to 72. The psychometric validity of the Korean version was supported [15].

#### Health literacy

Health literacy was measured with the Newest Vital Sign [16]. Patients are asked six questions about the "nutrition facts" label from a pint of ice cream. The number of correct responses is summed to produce a total score ranging from 0 to 6. Scores below four indicate limited to possibly limited health literacy.

#### Depressive symptoms

The nine-item Patient Health Questionnaire was used to measure the levels of depressive symptoms [17]. Patients were asked to rate their depressed moods over 2 weeks on a 4-point Likert scale (0–3). The sum of each item yields a total score, with higher scores indicating higher levels of depressive symptoms. A total score of 10 or greater indicates clinically significant depressive symptoms [17]. The validity of the Korean version was supported in a previous study [18].

#### Anxiety

The anxiety subscale of the Brief Symptom Inventory-18 was used to measure anxiety [19]. This subscale consists of six items based on a 5-point Likert scale (0–4). Total scores were the average of the scores of the six items, with higher scores indicating a higher level of anxiety. The psychometric soundness of the Korean version of the Brief Symptom Inventory-18 was supported in a previous study [20].

#### Sociodemographic information

Sociodemographic information (e.g., age, family history of hypertension) was obtained based on self-report. Height and weight were measured by trained research nurses using the standard clinical procedures (e.g., removing heavy clothes and shoes). Body mass index was calculated by weight in kilograms divided by height in meters. A medical record review for each participant was performed to collect the laboratory data (e.g., lipid panel).

### Study procedures

After obtaining ethical approval from the Institutional Review Board at the study site, eligible patients were referred to the researchers by their cardiologists. A written signed informed consent was obtained from each patient who received a thorough explanation of the study and voluntarily agreed to participate.

At baseline, automated office BP was taken using the standard procedure, and patients completed the questionnaires (e.g., sociodemographic information and health literacy). They also received verbal education, a booklet highlighting the importance of home BP monitoring at least twice daily, and recommended lifestyle modifications. They were also provided a contact number. Patients were instructed to stop their BP medications after the baseline data collection of this study, which was informed to their cardiologist.

Patients were followed up 1, 3, and 6 months after the baseline. Three automated office BP readings were taken. They also completed the questionnaire about their adherence to self-care activities for hypertension at each visit. Reasons for withdrawal from the study were also collected.

After the 6-month follow-up, participants were invited to participate in a semistructured, in-depth interview to elicit information about their experience with antihypertensive drug discontinuation (e.g., what changes did you make after drug discontinuation?). The interviews were conducted in person in a private space and lasted 20 to 50 min. Audio-recorded interviews were transcribed verbatim. The transcribed interviews were summarized to describe the experience of discontinuing antihypertensive medications and the reasons for withdrawal from the study, if applicable. Six patients participated in the interviews; two in the normotensive group, two in the hypertensive group, and two who withdrew from the study.

### Statistical analyses

To describe the success rate of antihypertensive medication discontinuation over 6 months, we used the Kaplan–Meier curve of time to have elevated office BP based on the preset criteria (i.e., above 140/90 mmHg on two consecutive visits, above 160/100 mmHg on any time visit, or physicians’ prescription for antihypertensive medications). The survival probability was summarized at 1, 3, and 6 months. The linear mixed model with repeated measures was used to compare changes in office BP readings over 6 months between the normotensive and hypertensive groups. Time was introduced as the repeated effect, and a compound symmetry covariance matrix handled dependencies in the data. Explanatory factors included the two BP status groups (normotensive and hypertensive groups), time, and the interaction between the BP status groups and time.

To compare the sample characteristics between the normotensive and hypertensive groups, the Mann–Whitney U-test (an alternative nonparametric test to an independent t-test), chi-square test, or Fisher exact test was used as appropriate. The linear mixed model with repeated measures was used to compare changes in adherence to self-care activities for hypertension over 6 months. In addition to comparing the characteristics between patients who dropped out of the study and those who did not, the Mann–Whitney U-test, chi-square test, or Fisher exact test was used as appropriate. A *P*-value of ≤ 0.10 was considered statistically significant for all analyses because of the study's exploratory nature. Statistical analyses were performed using SAS ver. 9.4 (SAS Institute Inc., Cary, NC, USA).

## Results

### Baseline sample characteristics

**Fig. 1:**
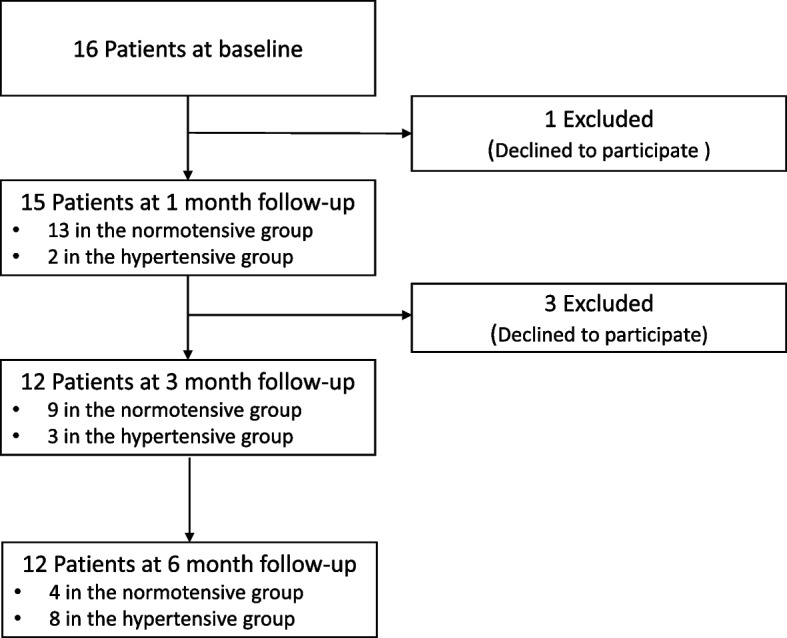
Study enrollment and hypertension status flow chart

A total of 16 patients with hypertension participated in this study. At 1, 3, and 6 months, 15, 12, and 12 patients remained in this study (Fig. 1). The mean ± standard deviation (SD) age was 41 years (range, 26–55 years) (Table 1). The majority of the patients were female (62.5%), had received at least a high school education (81.2%), and had a family history of hypertension (81.3%). Three patients (18.8%) had a body mass index above 25 kg/m^2^. All patients had taken angiotensin II receptor blockers, except one patient taking a calcium channel blocker. On average, participants had adequate health literacy and reported no clinically significant depressive symptoms.

**Table 1:** Baseline sample characteristics

Characteristic	Total (*n* = 16)	Normotensive (*n* = 8)	Hypertensive (*n* = 8)	*P*-value
Age (yr)	41.4 ± 9.3	42.0 ± 11.9	40.8 ± 6.6	0.598
Female sex	10 (62.5)	5 (62.5)	5 (62.5)	> 0.999
Lived with someone	13 (81.3)	7 (87.5)	6 (75.0)	> 0.999
Below high school education	3 (18.8)	2 (25.0)	1 (12.5)	> 0.999
Employed	9 (56.3)	5 (62.5)	4 (50.0)	> 0.999
Body mass index (kg/m^2^)	23.7 ± 2.8	23.5 ± 2.1	23.9 ± 3.5	0.753
Family history of high BP	13 (81.3)	7 (87.5)	6 (75.0)	> 0.999
Systolic BP at baseline	125.5 ± 8.6	122.8 ± 9.1	128.1 ± 7.6	0.155
Diastolic BP at baseline	83.0 ± 10.5	78.9 ± 11.7	87.0 ± 7.8	0.141
Time since hypertension diagnosis (mo)	68.6 ± 46.0	68.6 ± 50.7	68.6 ± 44.3	0.916
Antihypertensive medications				
Angiotensin II receptor blocker	15 (93.8)	8 (100)	7 (87.5)	0.182
Calcium channel blocker	1 (6.3)	0	1 (12.5)	> 0.999
Total cholesterol	192.9 ± 26.1	186.3 ± 12.8	197.2 ± 32.2	0.443
Triglycerides	97.5 ± 39.5	74.6 ± 11.5	120.4 ± 45.0	0.021
Low-density lipoprotein	109.1 ± 22.1	110.5 ± 14.7	107.7 ± 29.2	0.749
High-density lipoprotein	57.2 ± 12.4	62.0 ± 13.1	52.4 ± 10.6	0.159
Psychological factor				
Anxiety	0.5 ± 0.5	0.6 ± 0.6	0.4 ± 0.4	0.489
Depressive symptoms	2.1 ± 2.5	2.4 ± 2.4	1.9 ± 2.7	0.386
Health literacy	4.3 ± 1.6	4.5 ± 1.9	4.1 ± 1.5	0.513
Hypertension self-care				
At baseline	47.0 ± 8.1	47.8 ± 7.4	46.3 ± 9.2	0.713
At 1 month (*n* = 13)^a)^	52.9 ± 8.5	53.2 ± 7.6	52.8 ± 9.6	0.768
At 3 months (*n* = 12)	50.3 ± 9.2	49.3 ± 6.6	50.9 ± 10.6	0.734
At 6 months (*n* = 12)	51.3 ± 5.6	51.8 ± 6.2	51.0 ± 5.8	0.864

Data are presented as mean ± standard deviation or number (%)

*BP* blood pressure

^a^^)^At 1 month, two patients took their BP without completing the questionnaire

The average ± SD score of self-care adherence for hypertension at baseline was 47.0 ± 8.1, with possible scores ranging from 18 to 72. The self-care activities that were the least adherent were reading food labels to check for salt content, drinking in moderation, checking food labels for saturated fat or trans fat, and monitoring BP at home, in that order. In that order, the most adherent self-care activities were regular office visits, smoking cessation, and reducing calories from fat.

### Change in blood pressure

**Fig. 2:**
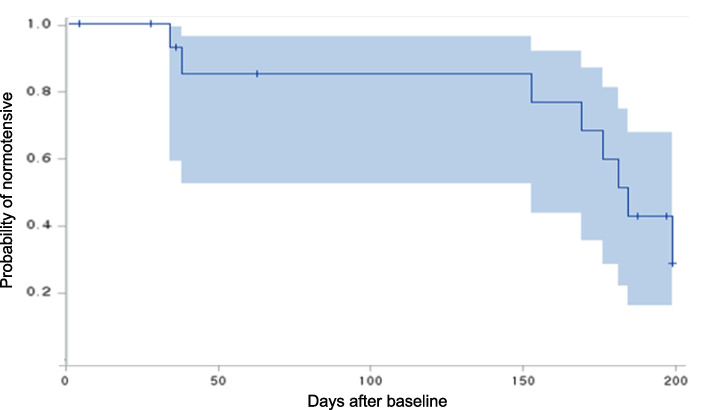
Survival plots of 16 participants’ normotensive status over 6 months. Blue areas indicate 95% confidence intervals

Patients were followed over 6 months (mean, 151 days; range, 5–195 days). The probability of normotensive status was 1.00 at 30 days, 0.85 at 90 days, 0.51 at 180 days, and 0.28 at 195 days. A relatively large decrease in the probability of normotensive status was found between 150 and 195 days after the baseline, with the likelihood of normotensive status of 0.77 and 0.28 at each time point (Fig. 2). Over 6 months, of the eight patients (50%) met the criteria for successful discontinuation of the antihypertensive medication (i.e., normotensive group).

The average ± SD systolic and diastolic BPs at baseline were 125.4 ± 8.6 and 82.9 ± 10.5 mmHg, respectively. The mean ± SD changes of systolic and diastolic BP levels from 6 months to baseline were 13.3 ± 8.8 and 10.1 ± 7.5 mmHg, respectively.

**Fig. 3:**
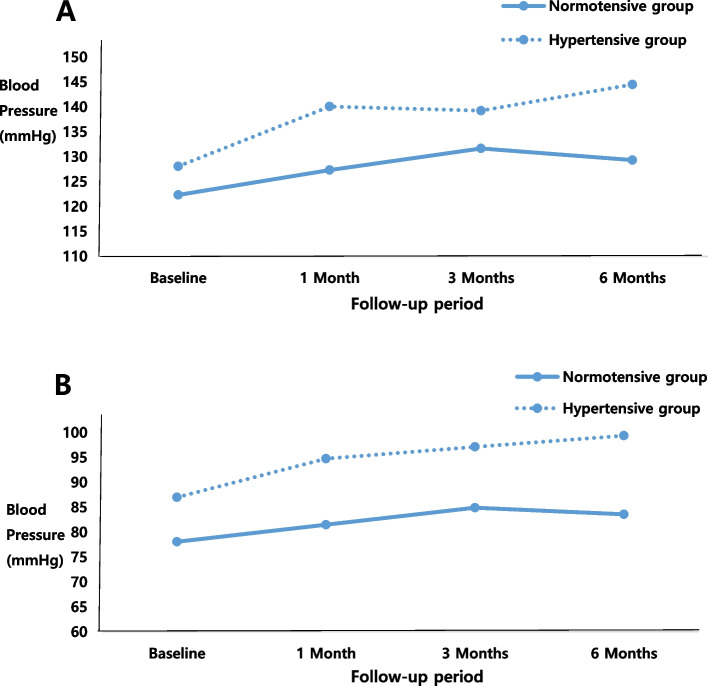
Changes in (**A**) systolic and (**B**) diastolic blood pressure over 6 months using 12 patients who completed the 6-month follow-up

Regarding the changes in the mean systolic BP by BP status groups, there were no time and group interaction effects (*P* = 0.160). However, there were significant main effects of time and groups (both *P* < 0.05), indicating substantial increases in the average systolic BP over time regardless of the group and significant differences in the average systolic BP between normotensive and hypertensive groups (Fig. 3). The mean diastolic BP was different between the normotensive and hypertensive groups, and a nonsignificant time and group interaction effect was found. However, significant main products of time and group were found in diastolic BP (both *P* < 0.05) (Fig. 3).

### Comparison of sample characteristics and changes in self-care adherence between the blood pressure status groups

There were no significant differences in demographic, clinical, and psychological status, except for triglyceride levels (Table 1). Patients in the normotensive group were likelier to have lower triglyceride levels than those in the hypertensive group (*P* = 0.028). After excluding the patients who withdrew from the study, the sample characteristics at baseline were compared between patients in normotensive and hypertensive groups, and there were no differences between the two groups.

Regarding changes in self-care adherence between the normotensive and hypertensive groups, there was a significant main effect of time, but not the main effect of group and the interaction effect between time and group. In other words, there was an increase in self-care adherence over 6 months for both groups (*P* = 0.06).

### Patient experiences related to discontinuing antihypertensive medications

Four patients in the normotensive (one male and one female) and hypertensive groups (one male and one female) were interviewed. All the patients expressed that they were satisfied with an opportunity to stop their antihypertensive medications because they believed that others stigmatized young to middle-aged adults taking antihypertensive medications. However, they all reported feeling anxious because they were worried about elevated BP after discontinuing their medication. One female patient said: “I am still anxious … so I always have the antihypertensive medication with me.”

All patients in the normotensive group made lifestyle changes throughout the 6 months. They reported that their BP had been under control, and their symptoms (e.g., fatigue) were improved. These positive experiences motivated them to maintain their lifestyle modifications, which resulted in enhancing their confidence in maintaining appropriate BP levels without medications. One female patient said she kept a detailed diary to record her daily BP readings and events that may have affected her BP. The log helped her identify major contributing factors to a rise in BP and helped her develop strategies to avoid or relieve the factors. Support from their family and physician also helped motivate them to change their lifestyle. A male patient said, “My physician gave me positive feedback on my BP readings with encouragement, which assured me that I do not need to take the medication anymore. This confidence makes me keep doing my lifestyle modifications.”

Patients in the hypertensive group did not make any particular changes in their lifestyle. One male patient said he had been following the recommended lifestyle already, so he did not find anything further to change. In contrast, one female patient, a homemaker, experienced difficulty exercising and eating healthy due to the coronavirus disease 2019 outbreak and the stress she experienced from it. Both patients felt stressed or anxious when they found their elevated BP readings. Although the female patient believed that not adhering to exercise and diet contributed to her rise in BP, she was not confident about making changes since she did not enjoy exercising and eating salads daily to cut down on calories. The male patient did not mention much about social support from his family. However, the female patient received negative feedback from her family, saying, “Why are you so bothered? Just take the antihypertensive medication.”

### Comparison in sample characteristics between patients who remained in the study and those who did not and their reasons for withdrawal

The retention rate of this study was 75%, with four patients dropping out of the study. There were no significant differences in the baseline sample characteristics, including the baseline systolic and diastolic BP readings between patients who remained in the research and those who dropped out. However, patients who remained in the study appeared to be younger and less anxious than those who withdrew from the study (40 years old vs. 47 years old, 0.42 vs. 0.79), although the relationships did not reach statistical significance. The four patients’ systolic and diastolic BP readings at their last follow-up visit were 127.8 ± 5.5 and 85.0 ± 3.4 mmHg, respectively.

Regarding the reasons for withdrawing from the study and deciding to resume their antihypertensive medications, one patient reported that his family was firmly against withdrawing antihypertensive medication. Two participants told significant concerns about their fluctuating BP despite adhering to the recommended lifestyle. The remaining patient felt she was not yet psychologically prepared to stop the medication.

Of the four patients, two patients (one male and one female) were interviewed. After stopping their medications, both patients had higher BP, which made their families worried. The parent of one male patient requested a second opinion from her primary care physician about her son’s decision to stop taking the antihypertensive medication. The physician had an unfavorable opinion. A female patient's spouse suggested that taking antihypertensive medication was acceptable and expected for individuals in their fifties. He reasoned that taking medications with stable BP readings was better than not taking medications with fluctuating BP readings.

## Discussion

Our study revealed that the participants’ BP was elevated after discontinuing the antihypertensive medications over 6 months. A notable reoccurrence of hypertension was developed 5 months after medication withdrawal. However, only a limited portion (less than 30%) of patients remained in normotensive status at the end of the follow-up period. In the patient interview, most patients expressed ambivalent feelings toward stopping medication (thrilled vs. worrying). Increase in psychological distress (e.g., anxiety) was the primary reason for withdrawal from this study although BP of the patients who withdrew from the study was under control. Our results suggest that clinicians must carefully select patients who could potentially benefit from an attempt to discontinue the antihypertensive medication. Moreover, continuous emotional support might be important to keep patients off-medication.

Antihypertensive medications are assumed to be a life-long treatment. However, a prolonged prescription of cardiovascular medications has been challenged because the length of time investigated in clinical trials on cardiovascular drugs is often limited [21]. Yuan et al. [22] found that the maximum duration of the randomized controlled trials for beta-blockers (e.g., carvedilol) ranged from 360 to 365 days. In reality, patients had been on those medications longer than 365 days [22]. Thus, there is a need for studies on drug withdrawal as well as the long-term effects of antihypertensive medications.

We found that 50% of the patients remained normotensive 180 days after drug withdrawal, similar to 38% in the systematic review by van der Wardt et al. [23]. However, the proportion of patients in the normotensive group in our study was higher than the proportion reported in the study by Sasamura et al. [13] in which similar thresholds for normotensive status were used (0% and 18% of the patients who previously took nifedipine and candesartan, respectively). This difference may be due to the inclusion criteria; most studies, including our study, included patients whose BP had been well-controlled [19], while the study by Sasamura et al. [13] enrolled the patients who had not been treated for BP for 1 year and with a family history of hypertension.

Most participants in our study had elevated BP after discontinuing antihypertensive medication, which is consistent with previous studies [11, 13, 24]. BPs in our sample continuously rose over 6 months. However, in other studies [11, 25] where the samples were older (mean age, 55–81 years) with a higher baseline BPs (systolic BP, 140.4–148.8 mmHg) compared to that of our study, patients’ BP was elevated 10 to 12 weeks after antihypertensive medication withdrawal but was maintained thereafter.

Researchers have studied whether demographic and clinical information is associated with normotensive status and consistently found that monotherapy of antihypertensive medications and lower baseline BP were predictors of normotensive status [23]. However, we did not find significant differences in baseline systolic and diastolic BP between the normotensive and hypertensive groups (123/79 mmHg vs. 128/87 mmHg, respectively). This may be due to the small sample size in our study to detect the differences.

Beyond the demographic and clinical factors potentially associated with hypertensive status, we first tested the psychological aspects and adherence to self-care activities for hypertension to the best of our ability. However, we did not find significant differences in these factors between the normotensive and hypertensive groups.

Surprisingly, patients’ levels of self-care were not associated with BP status. However, improving self-care activities is critical to maintaining BP when patients with hypertension are not on their antihypertensive medications. One possible reason for this non-significant relationship is that the changes patients made in self-care activities were not substantial enough to see benefits after discontinuing medications. Patients in both groups were more likely to engage in their self-care activities over the 6 months after stopping their medications even though their mean score of self-care activities was not optimal (47.0 at baseline and 51.3 at 6 months, out of possible scores of 18–72). The mean scores in our sample were lower than those of previous studies conducted on Asian patients with hypertension, including older Korean patients [15, 26].

The middle-range theory of self-care for chronic illness suggests that various factors affect self-care in patients with chronic disease, including skills, motivation, confidence, and support from others [27]. Our interview also showed consistent findings that social support from family and clinicians affected their motivation to engage in self-care activities. In addition, one patient in the normotensive group discussed skill development in the interview. She reported using a detailed diary to identify the primary source of BP changes in her daily life and develop strategies to minimize exposure to that source. Although the theory did not probe misperceptions about factors influencing self-care for chronic, one patient in the hypertensive group reported that his self-care activities were “ideal.” Previous studies have shown that patients’ perceptions of their adherence to self-care activities often differ from their actual engagement [28–30]. For example, 18.7% of community-dwelling adults overestimated their vegetable consumption [29]. The findings from our study and others highlight the need for strategies for patients who discontinue their antihypertensive medications by improving their skills and motivation, seeking social support, and helping them identify their actual levels of self-care activities.

Four of the 16 patients enrolled in our study withdrew from the study even though their BP levels were not considered hypertensive. The most common reason for withdrawal from the study was psychological distress (e.g., anxiety). Although anxiety levels were not statistically different between patients who remained in the research and those who did not, anxiety was somewhat lower in patients who remained in the study compared to those who did not. A similar finding was observed in previous studies [7, 24]; Buranakitjaroen et al. [24] found that about 10% of the patients who stopped their antihypertensive medications self-medicated by supplementing their antihypertensive medications due to anxiety or feeling unwell. Clinicians need to pay attention to patients' psychological status when deprescribing antihypertensive medication, which is one of the principles suggested by Coe et al. [31]. One patient reported that the family’s concern about medication withdrawal was a primary reason to withdraw from the study. As social support from family members is essential to behavior changes [27], it can be beneficial to involve family members when discussing the possibility of the medication withdrawal.

There are limitations to be noted in our study. Our study included a limited number of patients recruited from one tertiary medical center, which limits the internal and external validity of our findings. However, this study was a pilot study to calculate the effect size and examine the feasibility of a future large-scale study. The retention rate of our study was 75%, which is smaller than that of previous studies [13, 24]. However, we performed linear mixed modeling to use all available data for each patient. The treatment periods for taking antihypertensive medication were not collected so that it was not possible to examine the relationship between treatment periods and BP control.

## Conclusions

Our study showed that a small portion of the young and middle-aged adults with hypertension maintained normotensive status after discontinuing their antihypertensive medication over 6 months. Although we could not identify factors associated with success in maintaining BP over 6 months, our results imply that careful selection of eligible patients can increase the success of the withdrawal of antihypertensive medications. The primary reason for withdrawal from our study was fear of stopping medications, which highlights the importance of considering patients’ psychological status. Thus, clinicians need to spend sufficient time with patients to plan the withdrawal process (e.g., providing information on dealing with potential withdrawal symptoms and fear) so patients are prepared to stop their medications.

## Data Availability

The data supporting this study's findings are available from the corresponding author upon reasonable request.

## Abbreviations

BP: Blood pressure
SD: Standard deviation
STAR CAST: Short treatment with the angiotensin receptor blocker candesartan surveyed by telemedicine
